# Correction: Atorvastatin Improves Survival in Septic Rats: Effect on Tissue Inflammatory Pathway and on Insulin Signaling

**DOI:** 10.1371/journal.pone.0118383

**Published:** 2015-03-03

**Authors:** The PLOS ONE Staff

Several figures in the article included the wrong blots. The authors apologize for these errors and are providing corrected figures as well as the underlying raw blots.

The p-JNK blots in Fig. 4B were inadvertently include from lanes in Figure 3D of the publication below:

Diabetes. 2011 Mar;60(3):784–96. doi: 10.2337/db09-1907.

Physical exercise reduces circulating lipopolysaccharide and TLR4 activation and improves insulin signaling in tissues of DIO rats.

Oliveira AG, Carvalho BM, Tobar N, Ropelle ER, Pauli JR, Bagarolli RA, Guadagnini D, Carvalheir JB, Saad MJ.

In Fig. 2B, 2C, 2G, 4D, 6G and 6I several images of the lower bands are misplaced. This affects the following panels:

Fig. 2B: beta actin blot

Fig. 2C: insulin receptor blot

Fig. 2G: beta actin blot

Fig. 4D: beta actin blot

Fig. 6G: beta actin blot

Fig. 6I: beta actin blot

In Fig. 4B, 4E, 4F, 4G, 4H, 5A and 5B several images of the upper bands are misplaced. This affects the following panels:

Fig. 4E: pcjun blot

Fig. 4F: pcjun blot

Fig. 4G: p-IRS1 serine 307 blot

Fig. 4H: p-IRS1 serine 307 blot

Fig. 5A: NFkappaB blot by the following one:

Fig. 5B: NFkappaB blot

**Fig 2:**
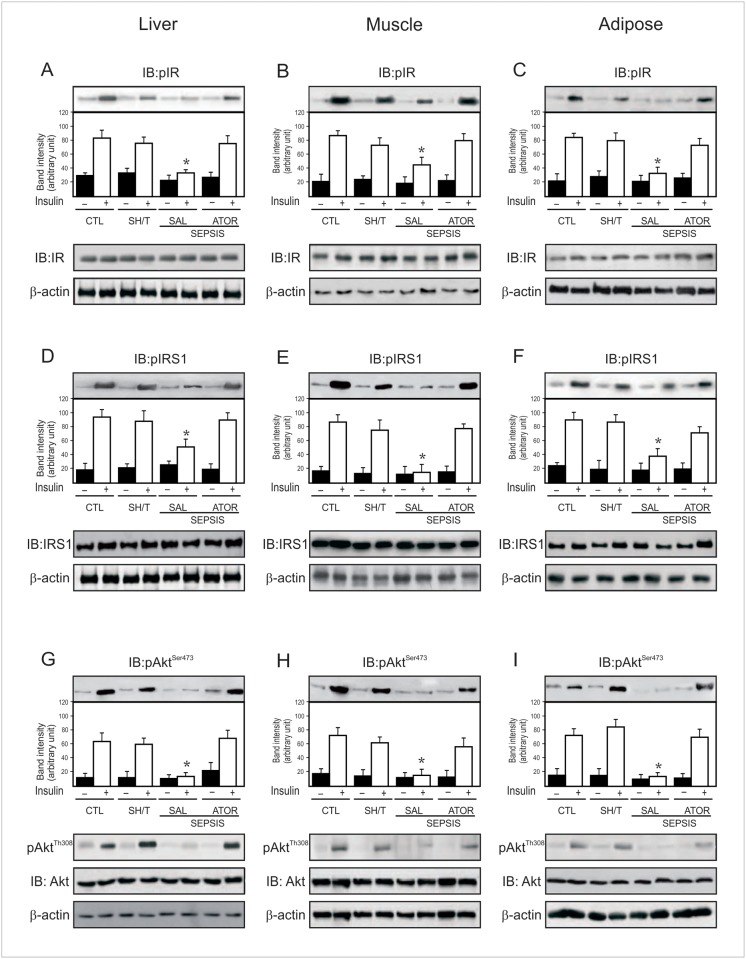
Effects of atorvastatin treatment on insulin signaling in the CLP rat. Representative blots show insulin-induced tyrosine phosphorylation of Insulin Receptor β (IRβ) in liver (A), muscle (B) and adipose (C) of sham and septic rats. Total protein expression of IRβ (A–C, lower panels). Insulin-induced tyrosine phosphorylation of Insulin Receptor Substrate 1 (IRS1) in liver (D), muscle (E) and adipose tissue (F) of sham and septic rats. Total protein expression of IRS1 (D–F, lower panels). Insulin-induced serine phosphorylation of Akt in liver (G), muscle (H) and adipose (I) of sham and septic rats. Insulin-induced threonine phosphorylation and total protein expression of Akt (G–I, lower panels). In this case, blots were stripped and reprobed with β-actin (A–I, lower panels) to confirm equal loading of proteins. Data are presented as means +/− S.E.M from 6–8 rats per group. *P<0.05 (Sepsis/Sal vs. all others groups). IB, immunoblot; CLT: Sham/Saline; ShT: Sham/Atorvastatin; SAL: saline; ATOR: atorvastatin.

**Fig 4:**
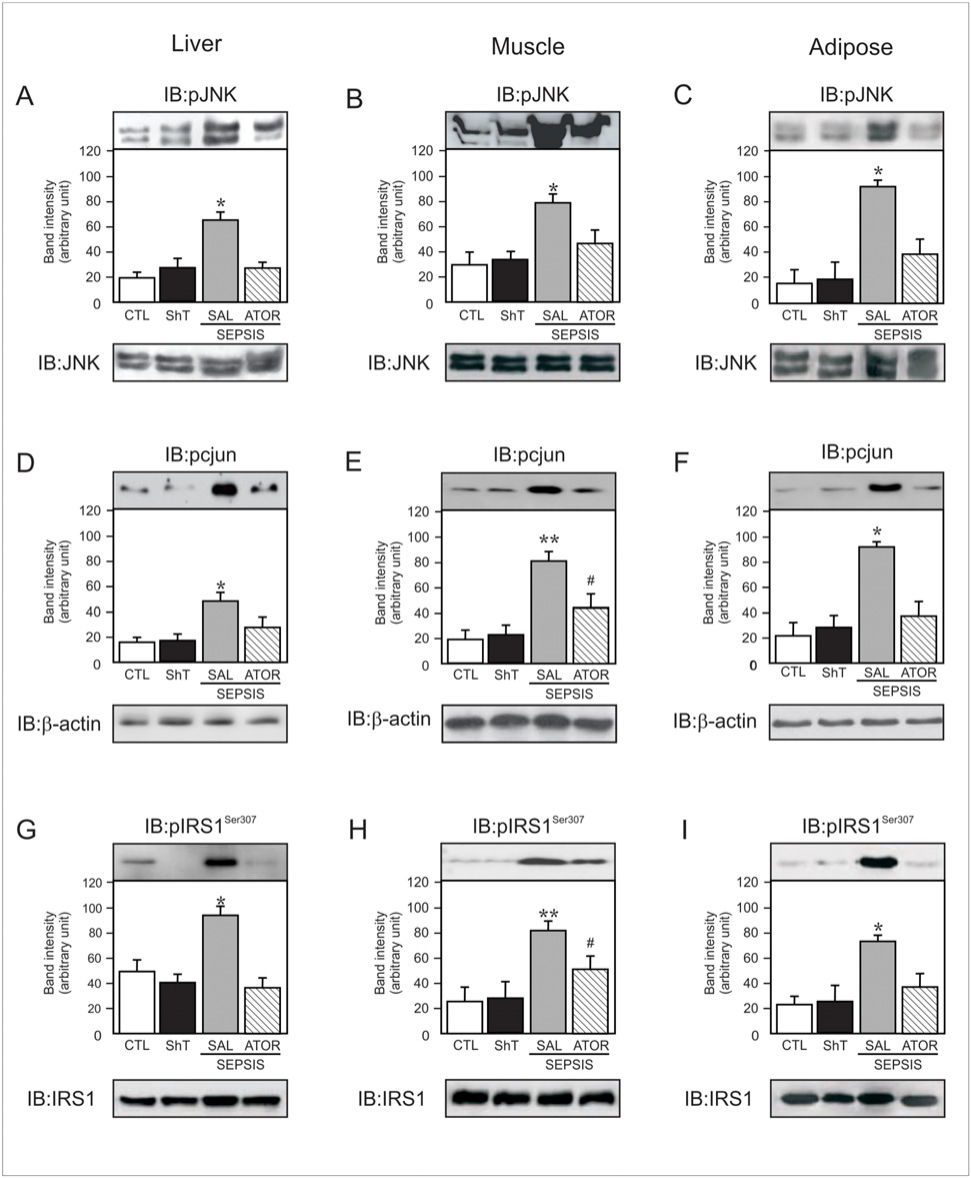
Representative blots show the JNK phosphorylation in liver (A), muscle (B) and adipose tissue (C) of sham and septic rats (upper panels). Total protein expression of JNK (A–C, lower panels). Phosphorylation of c-jun in liver (D), muscle (E) and adipose tissue (F) of sham and septic rats. Serine 307 Phosphorylation of IRS1 in liver (G), muscle (H) and adipose tissue (I) of sham and septic rats (upper panels). Total protein expression of IRS-1 (G–I, lower panels). Data are presented as means ± S.E.M from 6–8 rats per group. *P<0.05 (Sepsis/Sal vs. all others groups); **P<0.001 (Sepsis/Sal vs. control); #P<0.05 (Sepsis/Sal vs. Sepsis/Ator). IB, immunoblot; CLT: Sham/Saline; ShT: Sham/Atorvastatin; SAL: saline; ATOR: atorvastatin.

**Fig 5:**
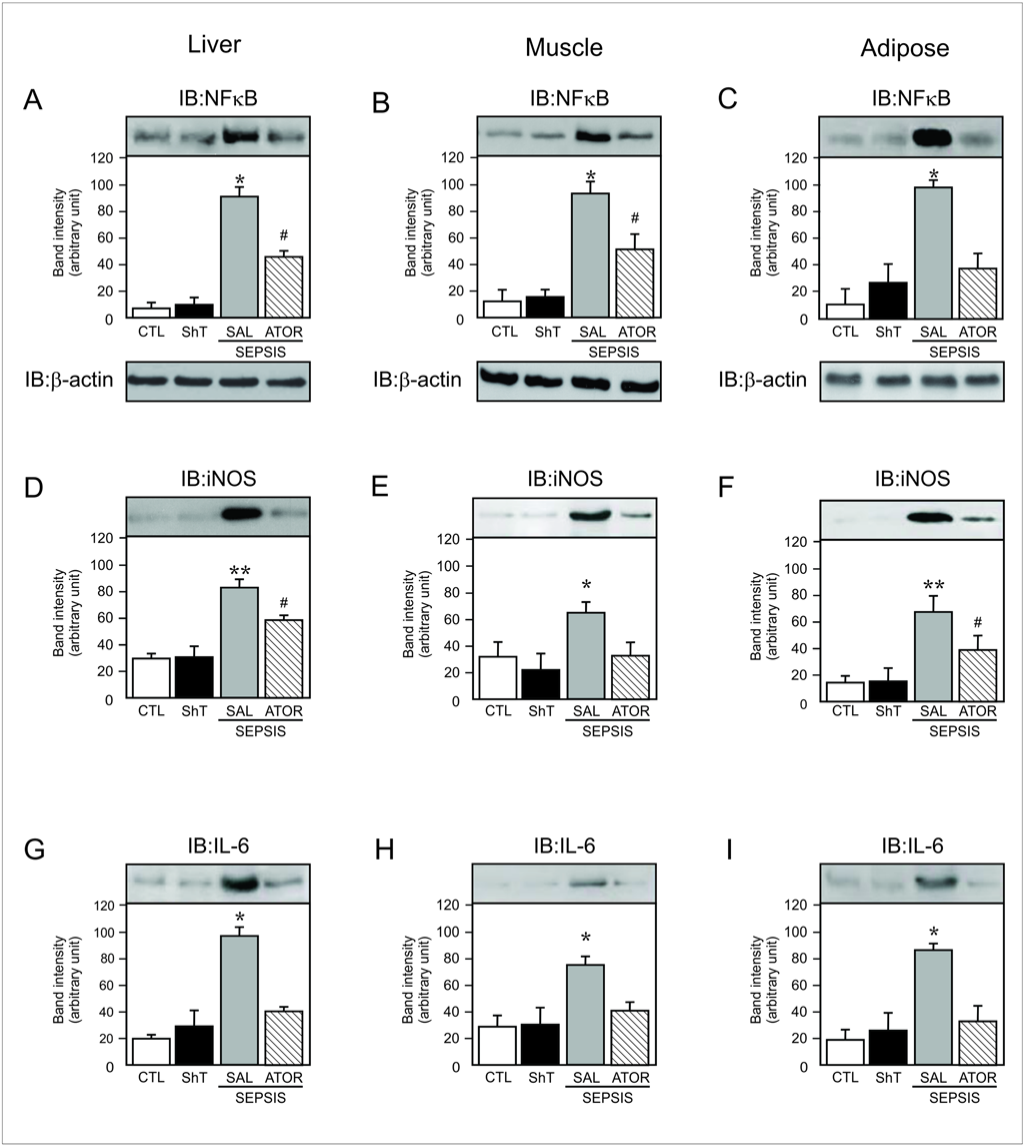
Representative blots show the NFkB activation in nuclear fractions of liver (A), muscle (B) and adipose tissue (C) of sham and septic rats. In this case blots were stripped and reprobed with actin (A–C, lower panels) to confirm equal loading of proteins. Tissue levels of iNOS (D–F) and IL-6 (G–I) expression in liver, muscle and adipose tissue of sham and septic rats. Data are presented as means ± S.E.M from 6–8 rats per group. *P<0.05 (Sepsis/Sal vs. all others groups); **P<0.001 (Sepsis/Sal vs. control); #P<0.05 (Sepsis/Sal vs. Sepsis/Ator). IB, immunoblot; CLT: Sham/Saline; ShT: Sham/Atorvastatin; SAL: saline; ATOR: atorvastatin.

**Fig 6:**
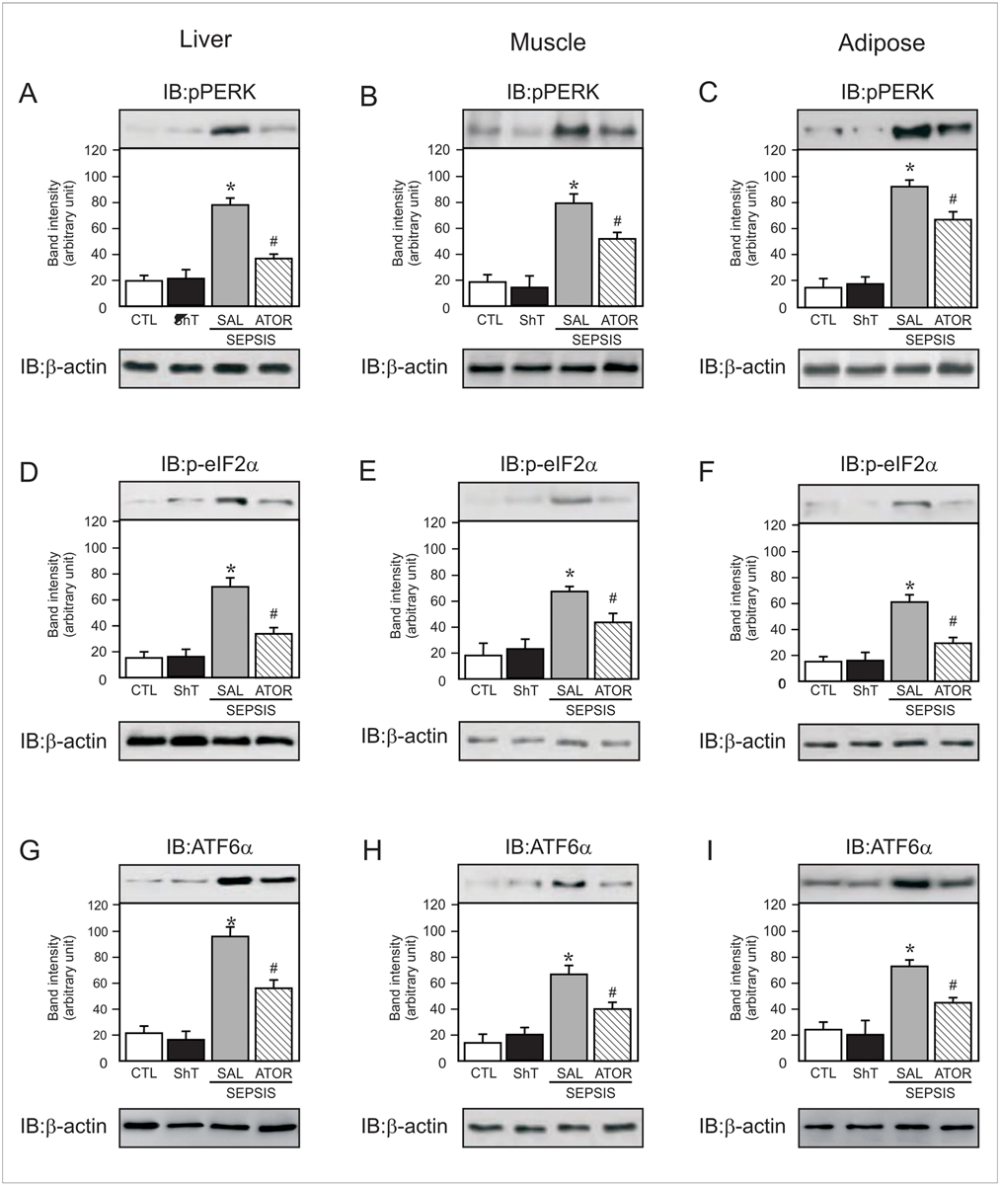
Representative blots show the PERK phosphorylation in liver (A), muscle (B) and adipose tissue (C) of sham and septic rats. eIF2α phosphorylation (D–F) and ATF6 (G–I) expression in liver, muscle and adipose tissue of sham and septic rats. In this case, blots were stripped and reprobed with actin (A–I, lower panels) to confirm equal loading of proteins. Data are presented as means ± S.E.M from 6–8 rats per group. *P<0.05 (Sepsis/Sal vs. all others groups); #P<0.05 (Sepsis/Sal vs. Sepsis/Ator). IB, immunoblot; CLT: Sham/Saline; ShT: Sham/Atorvastatin; SAL: saline; ATOR: atorvastatin.

## Supporting Information

S1 File Raw Blots. (DOCX)
