# Supplementary figures and images for: Correction: Atorvastatin Improves Survival in Septic Rats: Effect on Tissue Inflammatory Pathway and on Insulin Signaling
Source: PLoS One. 2015 Mar 3;10(3):e0118383. doi: 10.1371/journal.pone.0118383 (PMC4348178; doi:10.1371/journal.pone.0118383)

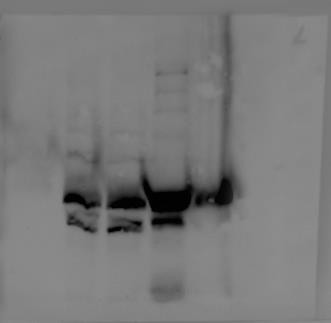


Blot 1 – 28/07/2009


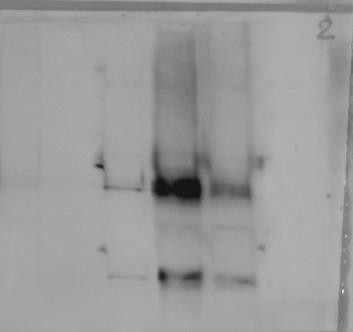


Blot 2- 08/07/2009


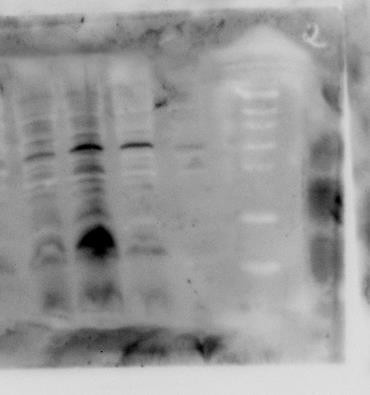


Blot 2- 01/07/2009


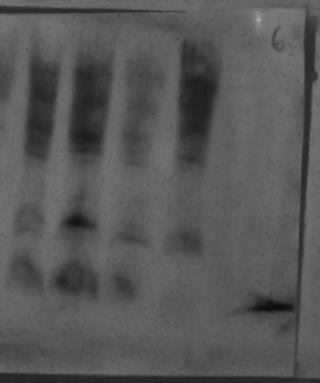


Image 6- 14/04/2009


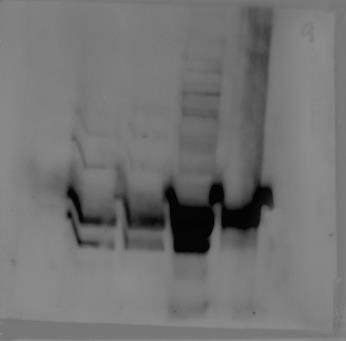


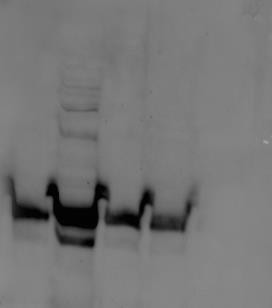


Blot 9- 01/09/2009

Blot 10 – 26/08/2009


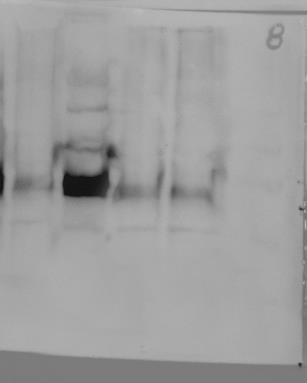


Blot 8 – 15/09/2009

Supplement: S1 File — (DOCX) [file pone.0118383.s001.docx]
